# Successful treatment of a patient with a ‘flow-through’ type II endoleak associated with an aberrant renal artery after endovascular aneurysm repair

**DOI:** 10.1093/jscr/rjad087

**Published:** 2023-03-11

**Authors:** Masaya Sano, Takuya Hashimoto, Mio Saito, Masaru Kimura, Masaya Miyazaki, Juno Deguchi

**Affiliations:** Department of Vascular Surgery, Saitama Medical Center, Saitama Medical University, Saitama, Japan; Department of Vascular Surgery, Saitama Medical Center, Saitama Medical University, Saitama, Japan; Department of Radiology, Saitama Medical Center, Saitama Medical University, Saitama, Japan; Department of Vascular Surgery, Saitama Medical Center, Saitama Medical University, Saitama, Japan; Department of Radiology, Saitama Medical Center, Saitama Medical University, Saitama, Japan; Department of Vascular Surgery, Saitama Medical Center, Saitama Medical University, Saitama, Japan

## Abstract

Identification and control of responsible feeding arteries are crucial in treating type II endoleaks after endovascular aortic repair (EVAR). A 78-year-old female patient required management of a type II endoleak 8 years after EVAR. A persistent endoleak from the inferior mesenteric artery (IMA) enlarged the size of an aneurysm sac. Sac angiography from the IMA revealed a flow-through endoleak from the IMA to an aberrant renal artery (ARA). After coil embolization of the ARA through the sac together with the IMA, the sac shrank. Control of flow-through vessels may be essential for managing post-EVAR enlargement due to type II endoleaks.

## INTRODUCTION

Endovascular aneurysm repair (EVAR) has become the standard treatment for abdominal aortic aneurysms (AAAs). Although several randomized studies have demonstrated a lower rate of perioperative mortality and complications, compared with open repair, EVAR has problems with long-term performance [1, 2]. A type II endoleak, which is caused by collateral blood flow to an aneurysm sac, is one of those problems. Although type II endoleaks do not cause adverse events in the short term, persistent type II endoleaks may result in sac enlargement, leading to aneurysm rupture. According to previous studies, risk factors of persistent type II endoleaks include advanced age, aneurysm diameter and patent branches from an aneurysm [3–5]. In addition, Muller-Wille *et al*. reported that ‘flow-through’ type II endoleaks that have feeding and drainage arteries are a high-risk factor for aneurysm enlargement [6].

Here we present a case of aneurysm sac enlargement after EVAR due to a type II endoleak from the inferior mesenteric artery (IMA) with the patient’s consent. After sac angiography from the IMA, we identified the ‘flow-through’ endoleak via the sac from the IMA to an aberrant renal artery (ARA). We successfully embolized the ARA through the sac and then the IMA with the coils, and the sac aneurysm decreased in size after treatment. No complications, such as renal deterioration, were observed. Identification and control of flow-through vessels of the sac may be essential for managing post-EVAR enlargement due to type II endoleaks.

## CASE REPORT

**Figure 1:**
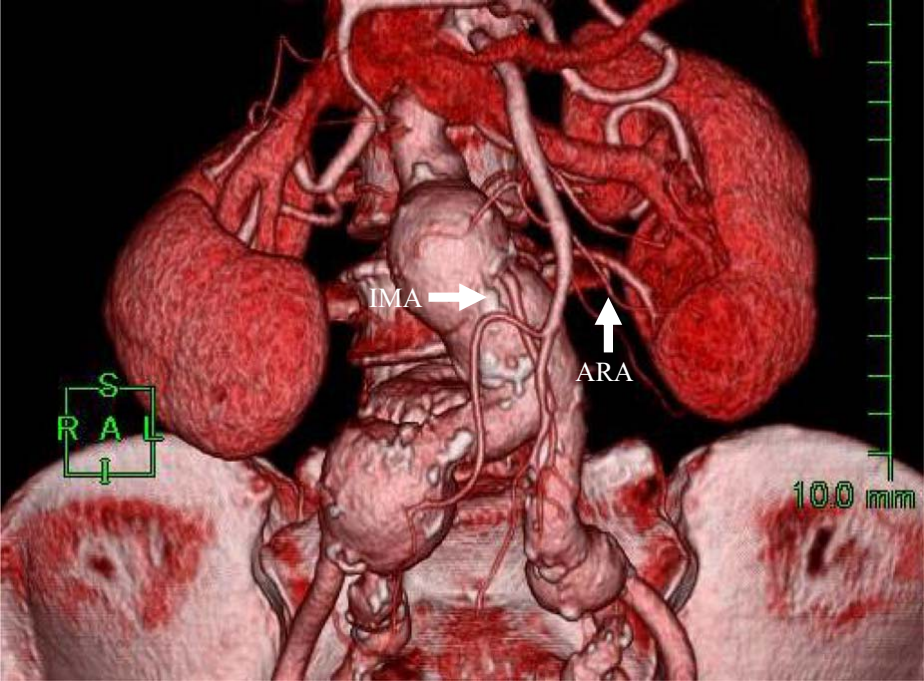
Preoperative CT angiography revealed that the IMA and ARA branched closely from the sac.

A 70-year-old female with a medical history of hypertension and dyslipidemia underwent EVAR for a 37 mm right common iliac artery aneurysm (CIAA). Preoperative imaging revealed that she also had small AAA (35 mm) and left CIAA (24 mm) (Fig. 1). In addition, she had a patent IMA and left ARA from the AAA sac. Stent-grafts were deployed in the right CIAA with right internal iliac artery embolization and the small AAA covering the IMA and the left ARA without embolization. A flared stent-graft leg was placed in the left CIAA at that time. Although postoperative CT showed a type II endoleak from the IMA, no enlargement of the AAA was observed by annual CT or ultrasound examination for 4 years after EVAR. However, during the next 3 years, the AAA and the left CIAA gradually enlarged to 50 mm and 35 mm, respectively.

**Figure 2:**
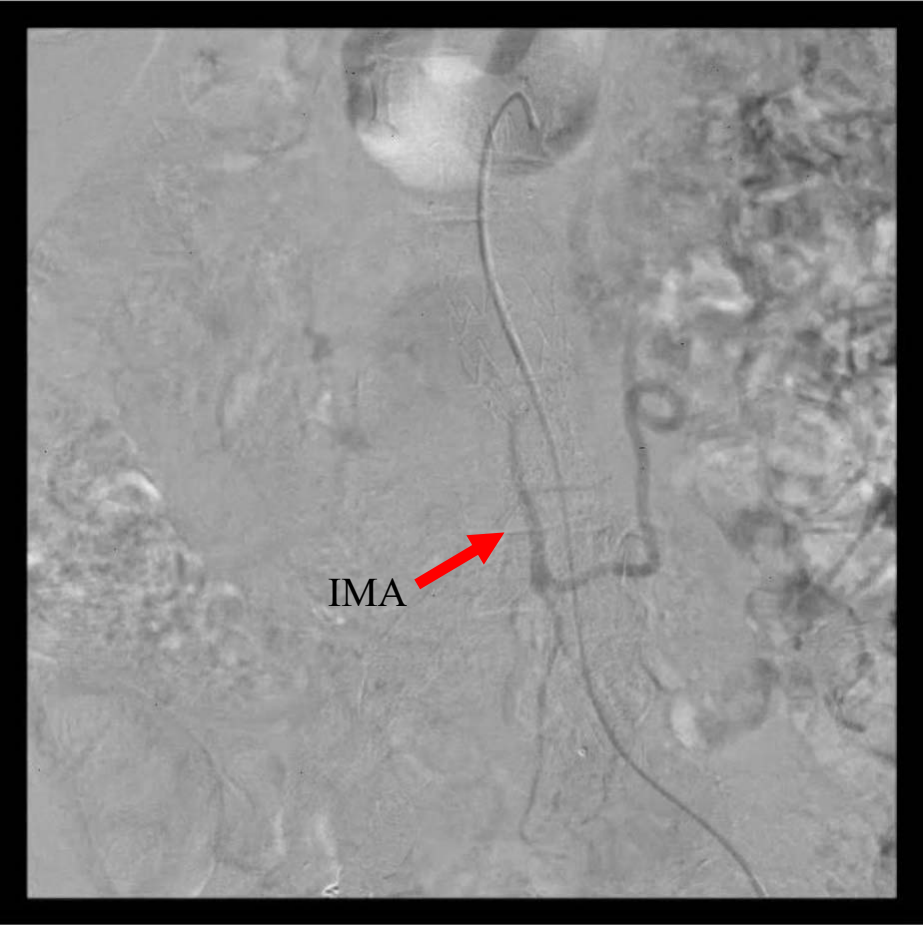
Angiography 8 years after EVAR demonstrated a type II endoleak from the IMA.

**Figure 3:**
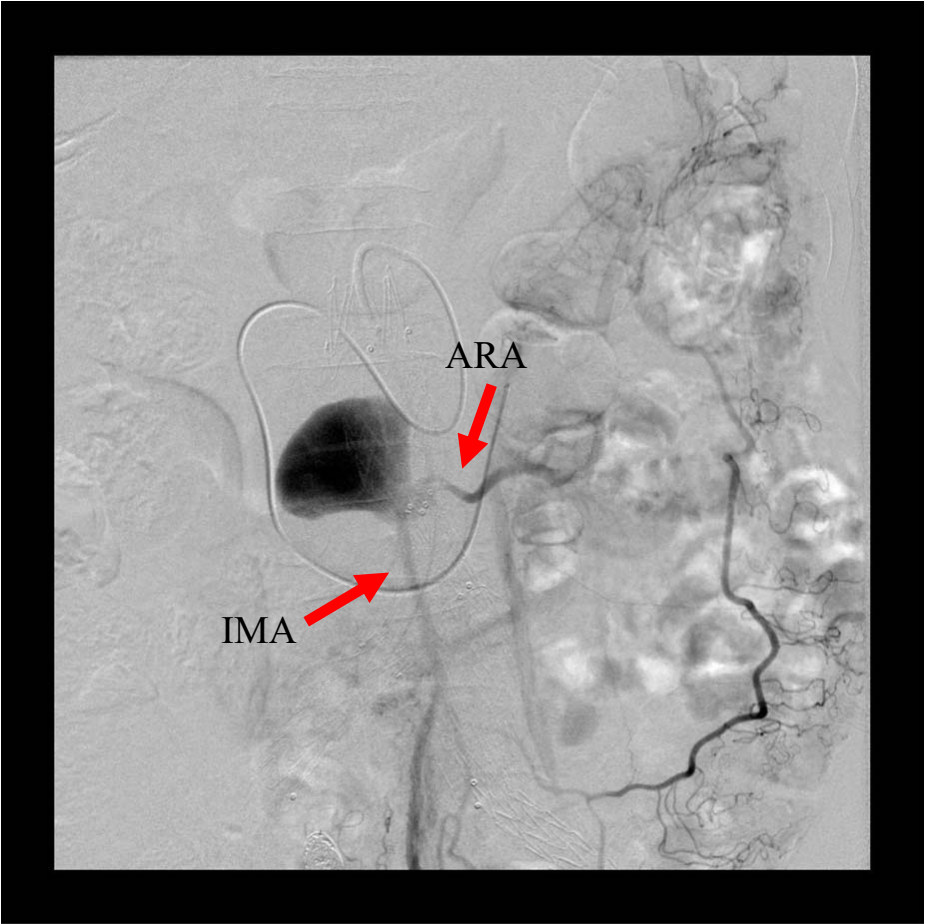
Sac angiography from the IMA via Riolan’s arcade showed a flow-through endoleak to the ARA as a drainage artery.

**Figure 4:**
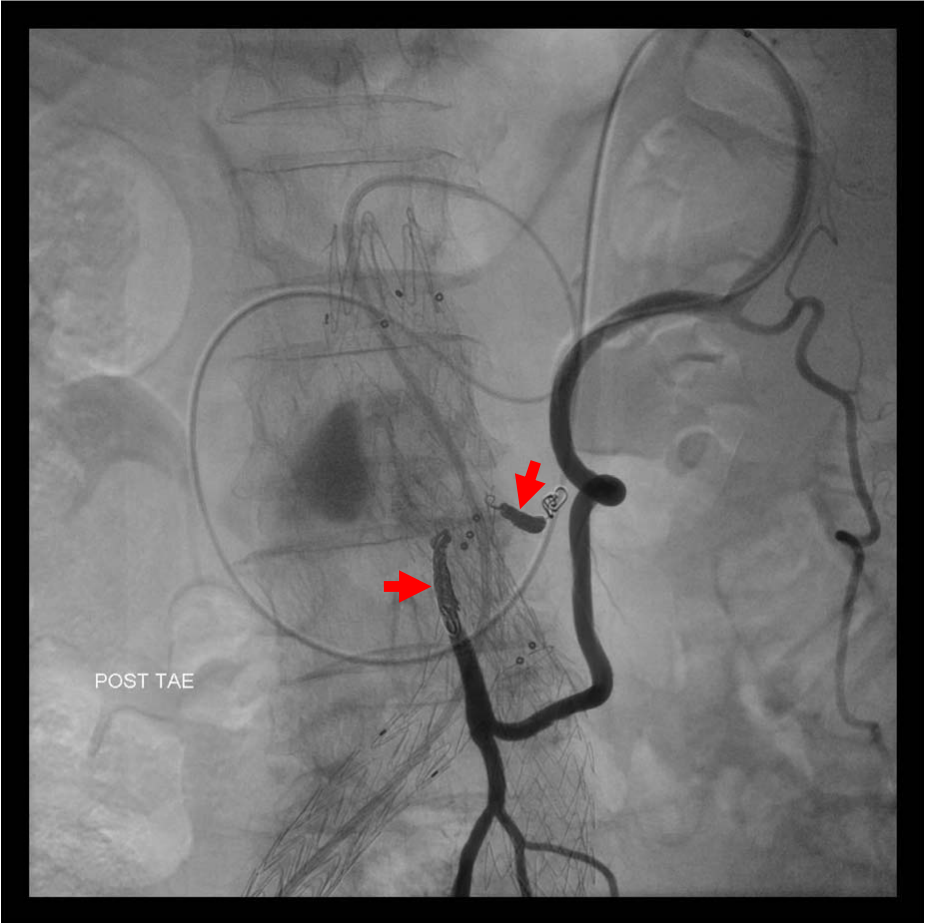
Coil embolization of the IMA and ARA was performed successfully via Riolan’s arcade.

**Figure 5:**
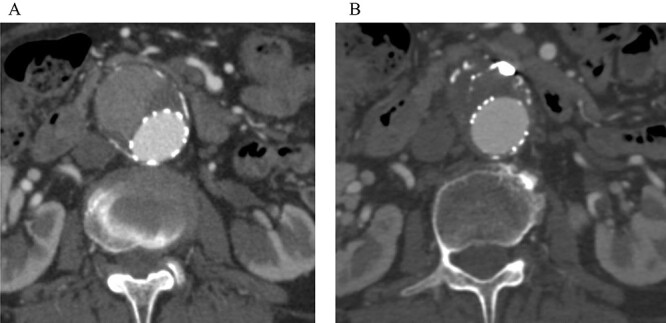
Post-embolization CT demonstrated that the sac shrank with no evidence of a continuing endoleak. (**A**) Pre-embolization CT showed a contrast effect in the sac. (**B**) Post-embolization CT showed no contrast effect in the sac.

Angiography for decision-making revealed a type II endoleak from the IMA to the AAA, but no other visible endoleak (Fig. 2). Based on these findings, we planned additional EVAR for left CIAA, which was thought to have enlarged in natural course, and IMA embolization for the type II endoleak associated with sac enlargement. A staged strategy was devised. IMA embolization was planned several days after EVAR. Sac angiography from the IMA via Riolan’s arcade showed a flow-through endoleak to the ARA as a drainage artery (Fig. 3). Based on this finding, we embolized the ARA and the IMA with coils via Riolan’s arcade (Fig. 4). Selection and embolization of the drainage artery were technically feasible because of the flow from the IMA to the ARA. No deterioration of renal function due to ARA embolization was observed. One year after treatment, a CT scan demonstrated that the sac shrank to 42 mm with no evidence of a continuing endoleak (Fig. 5).

## DISCUSSION

We presented a case involving a flow-through type II endoleak between the IMA and the ARA with aneurysm sac enlargement. After embolization of the ARA and the IMA via the Riolan’arcade, the endoleak disappeared, and the sac shrank.

Type II endoleaks are reported in up to 25% of cases immediately after EVAR [7]. However, half of them resolve spontaneously within 6 months. Around 25% of persistent type II endoleaks are reported to have sac enlargement, although ruptures are rare [8]. On the other hand, persistent type II endoleaks may induce type I endoleak via sac enlargement [9–11]. Although there is not enough evidence yet, the treatment is generally based on the sac size, growth (>5 mm) and symptoms. Trans-arterial embolization of the responsible branch is the most common method for treatment of type II endoleaks.

The most well-known risk factor for persistent type II endoleaks is patent branches from the sac, and the more branches from the sac, the higher the incidence of type II endoleaks [4]. In addition, Muller-Wille *et al*. reported a case of flow between two or more arteries via the sac, together with the diameter of the largest artery, as a risk factor for the sac enlargement [6].

ARAs are anatomical anomalies that are sometimes encountered when performing EVAR. Though the potential risks of covering them include renal infarction with deterioration of renal function and type II endoleaks, there is no evidence to recommend pre-emptive embolization. A previous systematic review reported that no significant change in renal function was found, and a few cases with type II endoleaks showed no significant difference in mortality or secondary reintervention [12].

In this case, the IMA and ARA branched closely from the sac, and their diameters were both large (IMA 3.7 mm, ARA 2.6 mm). We thought that continued flow from the IMA to the ARA via the sac led to sac enlargement. Embolization was successfully performed, and after treatment the sac shrank. No complications, such as renal deterioration, were observed.

Though very few cases of sac enlargement due to type II endoleaks involving an ARA have been reported [13], surgeons should be aware of the possibility of type II endoleaks involving the ARA when performing EVAR.

## CONFLICT OF INTEREST STATEMENT

None declared.

## FUNDING

None.

## AUTHORS' CONTRIBUTIONS

Design, M.S., T.H., M.K., J.D.; Data collection and analysis, M.S., T.H., M.S., M.K., M.M., J.D.; Writing the manuscript, M.S., T.H., J.D.
